# Physicians’ Mutual Benefit Association of Texas

**Published:** 1886-10

**Authors:** 


### SOCIETY NOTES.

### PHYSICIANS' MUTUAL BENEFIT ASSOCIATION OF TEXAS.

THE following members paid the assessment for the benefit of Certificate No. 89, (Sayers), after the list was published in our last issue: Drs. Y. D. Harrington, G. W. Gray, Jno. Inabnit, W. T. Richmond, T. J. McFarland.

Twenty-seven new members have joined since Dr. Sayers' death, July 2, and there are now, in good standing, 141; of whom 136 are liable for assessment No. 3, just sent out, for Certificate No. 134 (Dr. A. Welch, died Sept. 22), five having joined since.

BASTROP, Texas, Sept. 9, 1886.

F. E. DANIEL, M. D., Austin, Tex.:

*Dear Doctor*—Your favor of sixth inst. received with inclosed draft to Mrs. Sayers.

I presented your letter and the draft in person, she expressing her grateful appreciation of your kind interest in her distress. Inclosed find receipt, and I trust further acknowledgment may be made through your JOURNAL. With best wishes for yourself and *our* JOURNAL, I am, yours truly,

A. GARWOOD.

Received, Bastrop, Texas, of Dr. F. E. Daniel, Secretary Physicians' Mutual Benefit Association, of Texas, sight draft on Swenson & Sons, of New York, for $109, in payment of death benefit due Dr. D. Sayers' heirs, under provisions of Certificate No. 89. MRS. INDA SAYERS.

